# Performing laparoscopic sleeve gastrectomy in an obese patient with systemic lupus erythematosus undergoing long-term steroid therapy: a case report

**DOI:** 10.1186/s40792-019-0735-1

**Published:** 2019-10-29

**Authors:** Atsushi Gakuhara, Yasuhiro Miyazaki, Yukinori Kurokawa, Tsuyoshi Takahashi, Makoto Yamasaki, Tomoki Makino, Koji Tanaka, Kiyokazu Nakajima, Masaki Mori, Yuichiro Doki

**Affiliations:** 1 Department of Gastroenterological Surgery, Osaka University Graduate School of Medicine, 2-2, E-2, Yamadaoka, Suita City, Osaka, 565-0871 Japan; 2 Department of Surgery and Science, Graduate School of Medical Sciences, Kyushu University, 3-1-1, Maidashi, Higashi-ku, Fukuoka City, 812-8582 Japan

**Keywords:** Systemic lupus erythematosus, Bariatric surgery, Laparoscopic sleeve gastrectomy

## Abstract

**Background:**

Systemic lupus erythematosus (SLE), an autoimmune disease characterized by systemic inflammatory lesions, is often associated with obesity. Obesity aggravates symptoms of SLE; however, these symptoms can be improved by weight loss through diet therapy and bariatric surgery. However, there are only a few reports regarding the effectiveness of bariatric surgery in obese patients with SLE. Herein, we discuss the laparoscopic sleeve gastrectomy (LSG) performed in an obese patient with SLE while undergoing long-term steroid therapy.

**Case presentation:**

A 36-year-old female, suffering from SLE for 10 years with effects on the central nervous system, developed diabetes mellitus (DM) triggered by the steroid therapy for SLE. The patient was undergoing steroid therapy (6 mg/day) for SLE since a long time. For DM management, her HbA1c level was maintained at 7.4%. She was 158 cm tall and 91.6 kg in weight. Her body mass index was 36.7. She could not work and depended on welfare services. To improve her obesity and DM, physicians suggested that she should undergo bariatric surgery in our hospital. Eventually, she underwent LSG, which lasted for 185 min, with minimal blood loss and without complications. Her blood glucose level stabilized immediately after the surgery; hence, her antidiabetic medication was discontinued. She was discharged 8 days after surgery, and her weight decreased steadily. In the first year after surgery, her weight was 54.4 kg, and she had lost approximately 37 kg from her initial weight. Her steroid requirement had also reduced to 4 mg/day. Through weight loss, she could begin to work and became a part of society again.

**Conclusion:**

LSG was safely performed in an obese patient with SLE undergoing long-term steroid therapy. We noted substantial weight loss, improved DM condition, and reduced requirement of SLE therapy after surgery. Hence, surgical risks must be carefully examined before patients undergo bariatric surgery.

## Background

Systemic lupus erythematosus (SLE), an autoimmune disease characterized by systemic inflammatory lesions caused by tissue deposition of immune complexes such as DNA-anti-DNA antibodies, is often associated with obesity [1, 2]. Symptoms of SLE are worsened by obesity but can improve by weight loss through diet therapy [3]. Bariatric surgery is another effective way to reduce weight. However, only a few reports concerning the effectiveness of bariatric surgery on obese patients with SLE [4, 5]. Patients with SLE often undergo long-term steroid therapy, which poses a high surgical risk [6–8]. Herein, we report the case of an obese patient with SLE undergoing long-term steroid therapy in whom laparoscopic sleeve gastrectomy (LSG) was successfully performed.

## Case presentation

**Fig. 1:**
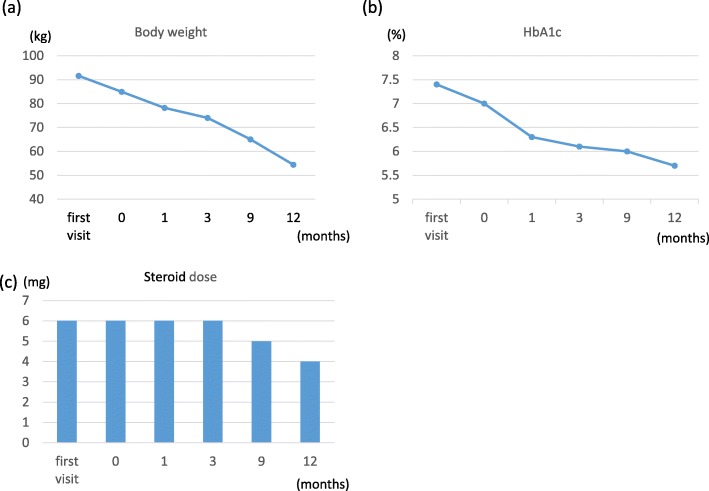
**a** Body weight change from baseline to 1 year after the surgery. **b** HbA1c change from baseline to 1 year after the surgery. **c** Steroid dose change from baseline to 1 year after the surgery

**Fig. 2:**
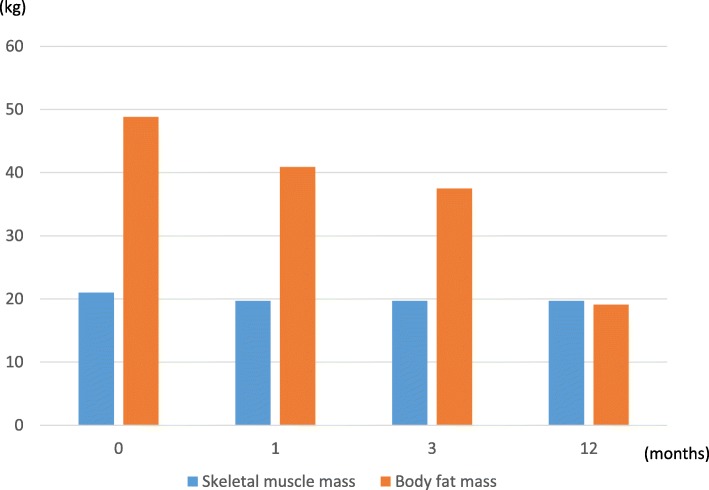
Body composition change from baseline to 1 year after surgery

A 36-year-old female, suffering from SLE since 10 years with effects on her central nervous system, developed diabetes mellitus (DM) 9 years ago, triggered by her long-term steroid therapy for SLE. She was undergoing steroid treatment (6 mg/day) for SLE at a different hospital. She was 158 cm tall and weighed 91.6 kg. Her body mass index was 36.7, indicating 3° higher obesity. To manage DM, she was treated with metformin, and her HbA1c was controlled at 7.4%. Serum immuno-reactive insulin (IRI) and C-peptide immunoreactivity (CPR) levels were 13.8 μU/ml and 2.5 ng/ml, respectively. Both the markers were in normal range. Total cholesterol (T-chol), triglyceride (TG), high-density lipoprotein cholesterol (HDL-C), and low-density lipoprotein cholesterol (LDL-C) levels were 191 mg/dL, 86 mg/dL, 41 mg/dL, and 126 mg/dL, respectively. Her dyslipidemia was controlled by administering atorvastatin. She had no hypertension as a complication of obesity. She was also treated with paroxetine hydrochloride hydrate, mianserin hydrochloride, and sodium valproate for steroid-induced depression. She could not work and depended on welfare services. To improve her obesity and DM, physicians suggested that she should undergo bariatric surgery in our hospital. She understood bariatric surgery well, and the symptoms of SLE were well controlled and stable, and she had no symptoms of central nervous system lupus. Anti-DNA and anti-Sm antibody levels were > 2.0 IU/ml and 2.5 U/ml, respectively. Both the SLE markers were in normal range. CH50, C3, and C4 levels were 53.8 U/ml, 144 mg/dL, and 26 mg/. All the SLE markers were in normal range, and SLE activity was well controlled as per laboratory data. She was given a diet instruction by her previous doctor but was unable to lose weight. Her obesity was considered to include an element of secondary obesity due to steroids. However, there were several studies reporting that patients with SLE who were obese were able to reduce their steroid dose along with reduction in their weight after bariatric surgery. Therefore, this case was judged to be an indication for bariatric surgery. Preoperative weight loss techniques were demonstrated at our outpatient clinic. She was treated with Mazindol and given diet instruction by a dietitian. She was able to lose 7 kg while continuing nutritional guidance for approximately 5 months. She was admitted in our hospital a week before the surgery, and her blood glucose was controlled. Thereafter, she underwent LSG, which lasted for 185 min, with a minimal blood loss and without complications. For the perioperative steroid cover, 200 mg of hydrocortisone sodium succinate was administered immediately before the surgery, and the maintenance dose (6 mg/day) was resumed on the first postoperative day. She was discharged on the eighth postoperative day without complications. Her blood glucose level stabilized immediately after the surgery; hence, her antidiabetic medication was stopped. After being discharged, she experienced gradual weight loss. After a year, she weighed 54.4 kg, losing approximately 37 kg, compared with her weight during the initial examination; additionally, rebound effects were not manifested (Fig. 1a). Body fat mass was also markedly reduced 1 year after the surgery compared with her preoperative status; meanwhile, her skeletal muscle mass was maintained (Fig. 2). Furthermore, SLE condition was stable, and her steroid requirement was reduced to 4 mg/day (Fig. 1c). After surgery, her blood glucose level had settled down; thus, her DM drug therapy was halted. Her DM improved up to 5.7% of HbA1c 1 year after the surgery (Fig. 1b). Her dyslipidemia was well controlled by atorvastatin. T-chol, TG, HDL-C, and LDL-C levels were 165 mg/dL, 43 mg/dL, 48 mg/dL, and 104 mg/dL, respectively. For her steroid-induced depression, paroxetine hydrochloride hydrate and mianserin hydrochloride were reduced by half, and sodium valproate medication was terminated 9 months after surgery. Through weight loss, she could begin to work and be part of the society again.

## Discussion

We were able to perform LSG safely for the obese patient with SLE undergoing long-term steroid therapy. Postoperatively, she had successfully lost weight, her DM improved, and her steroid dose was reduced. Ultimately, she became part of the society again.

The prevalence of SLE is higher in women than in men; of note, female population is six times larger than male population [9]. Regarding race, Asian and African populations are larger than Caucasian population [10]. SLE can be improved by administering NSAIDs, steroids, or immunosuppressive drugs; however, this condition is a refractory disease that subsides and relapses repeatedly. Long-term steroid use can lead to various unfavorable effects, such as cardiovascular lesions, DM, obesity, and infectious diseases, which lower the patient’s quality of life and prognosis [11, 12].

Regarding the relationship between SLE and obesity, two thirds of patients with SLE were overweight or obese [2]. In obese patients with SLE, functional ability and health-related quality of life are lower than that in normal weighing patients with SLE [13, 14]. In addition, the inflammatory marker is high [13]. Diet therapy had reportedly improved the SLE symptoms of a patient; hence, weight loss may be effective in ameliorating such symptoms in obese patients [3].

Bariatric surgery does not only promote weight reduction on severe obesity but also improve DM and metabolic syndromes [15]. Weight loss after bariatric surgery results in anti-inflammatory effects; in fact, bariatric surgery was performed for inflammatory bowel disease, and anti-inflammatory effects were manifested [16]. However, the effect of bariatric surgery on patients with SLE in Western Europe has been rarely reported [4, 5]. Corcelles et al. reported 1 gastric banding, 23 Roux-en-Y gastric bypass, 3 revisional surgery, and 3 LSG cases, with postoperative weight loss and possibility of therapeutic agent reduction. Bariatric surgery may be a possible treatment for SLE with obesity. The occurrence of anti-inflammatory effects due to weight loss may indicate the effectiveness of bariatric surgery for patients with SLE. However, the type of bariatric surgery that can yield optimal results is still unknown. Previous reports stated that silicon exposure causes a newly identified syndrome called autoimmune/inflammatory syndrome, which is induced by adjuvants and may subsequently develop into various systemic autoimmune diseases, such as SLE and rheumatoid arthritis; thus, this technique should be avoided [17, 18]. To our knowledge, our report is the first in Asia, and it shows that bariatric surgery for obese patients with SLE may be effective as well in Asians. Moreover, complications increase with respect to surgical risks for patients with autoimmune diseases, especially those who are using immunosuppressive drugs for a long time [6–8]. A steroid cover is also required in perioperative management; thus, we need to be cautious during perioperative management. In this case, by replacing corticosteroids on the day of surgery and restarting steroid therapy immediately after surgery, she showed a good postoperative course and no complications. Surgery indication should be decided after careful examination of a surgical risk.

## Conclusion

LSG was safely performed in such obese patient with SLE undergoing long-term steroid therapy. Postoperative weight loss was achieved, her DM improved, and her SLE therapy drugs were reduced. Thus, a careful examination on the surgical risks of patients is necessary before surgery and before selecting the appropriate procedure.

## Data Availability

Not applicable.

## Abbreviations

DM: Diabetes mellitus
LSG: Laparoscopic sleeve gastrectomy
SLE: Systemic lupus erythematosus
